# Supplementary material for: Intrinsic dichroism in amorphous and crystalline solids with helical light
Source: Nat Commun. 2024 Feb 14;15:1350. doi: 10.1038/s41467-024-45735-9 (PMC10867019; doi:10.1038/s41467-024-45735-9)
Supplement: Supplementary file 1 — Supplementary Information [file 41467_2024_45735_MOESM1_ESM.pdf]

Intrinsic dichroism in amorphous and crystalline solids with helical light

Ashish Jain[1,*,+], Jean-Luc Bégin[1,*,+], Paul Corkum[1], Ebrahim Karimi[1], Thomas Brabec[1], and Ravi Bhardwaj[1,*]

[1]Department of Physics, University of Ottawa, Ottawa, ON, K1N 6N5, Canada
[*]Corresponding Author. Ashish Jain, Jean-Luc Bégin, Ravi Bhardwaj. ajain067@uottawa.ca, jbegi038@uottawa.ca, ravi.bhardwaj@uottawa.ca
[+]These authors contributed equally to this work

Contents

1 Experimental setup 1
2 Crystal symmetry from transmission measurement 2
3 Tunability and scalability of HD(Type I) in crystals 4
4 Fluence Calculation 4
5 Differential absorption with circulary polarized Gaussian beam 5
6 Noise check: Transmission measurements in air in the absence of sample 5
7 Asymmetric Laguerre-Gaussian beams 6
8 Multiphoton assisted tunneling 7
9 Total MPAT transition rate for linear polarization 9
10 Supplementary References 10

1 Experimental setup

Transmission measurement were performed using a Ti: Sapphire laser amplifier system (45 fs, 800 nm pulses, pulse energy of 2.5 mJ). Figure S1 shows the schematic of our experimental setup. For each successive laser pulse, the energy was varied using a combination of half-wave plate (HWP) and polarizer. Light beams carrying orbital and/or spin angular momentum were generated and controlled by OAM/SAM unit consisting of a combination of quarter-wave plates (QWP), linear polarizer (LP) and a birefringent liquid crystal based phase plate called q-plate[1,2]. When an incident Gaussian beam propagates through the q-plate with a topological charge q, it acquires an OAM defined by $l = \pm 2q$ with a phase singularity (optical vortex). The singularity/null intensity region in the OAM beam was displaced by translating the q-plate, mounted on a x-,y-stages. Moving the q-plate with a step size of $250 \pm 10\mu$m translates to a displacement of the singularity by 300 ± 20nm at the focus.

For every laser shot, the transmitted light signal on the photodiode (PD2) was normalized with the incoming light signal on PD1, reflected off a glass plate positioned in the beam path. An aspheric objective lens (NA=0.3) was used to focus the femtosecond pulses to a spot size 2±0.2 μm into solid samples mounted on a three-axis translation stage. A second aspheric objective with the same NA collected and colliminated the transmitted light onto a photodiode (PD2), positioned immediately after the objective. For each experiment, laser was focussed in the middle of solid sample with typical dimensions $10 \times 10 \times 1$ mm. This was achieved by accurately finding the surface of the sample using back-reflected light that was imaged by a CCD camera. The signals generated by PD1 and PD2 were stretched by an electronic pulse stretcher, discretized and recorded by a data acquisition card. The incident pulse energies were measured before the objective. During the measurement, for every laser shot, the sample was translated by 5μm to irradiate fresh sample. A single-shot auto-correlator (not shown) continuously monitored the pulse duration. The pulse duration at the interaction region was about 100fs.

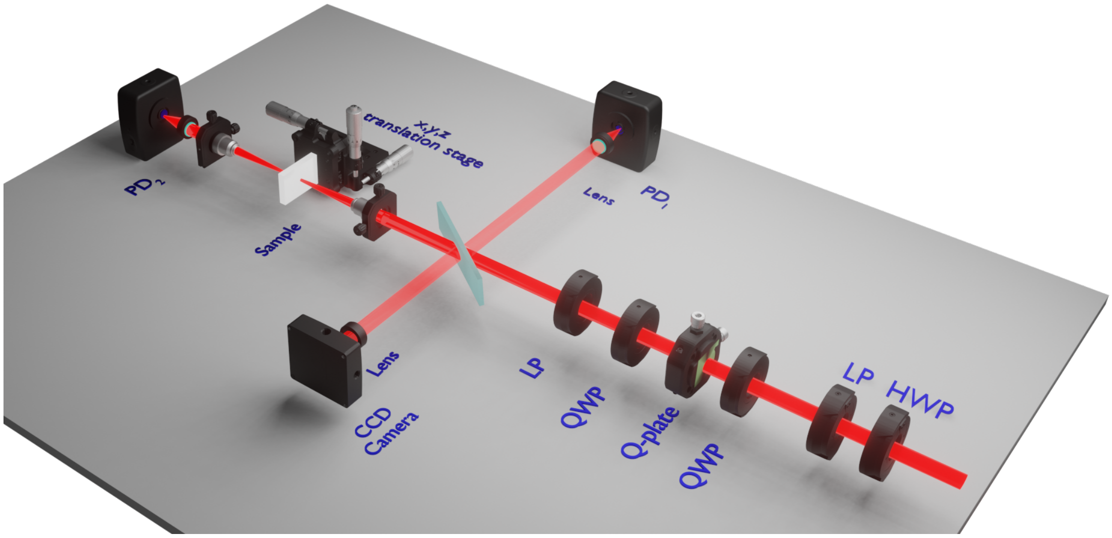

FIG. S 1. Schematic of the experimental setup. The power control consists of a combination of linear polarizer (LP) and half-wave plate (HWP). The OAM and SAM control consists of a combination of quarter-wave plates (QWPs), a birefringent liquid-crystal phase plate (q-plate) and LP (a third QWP can be used to generate elliptically polarized OAM light). Photodiode PD1 (PD2) monitors the incident (transmitted) light. The CCD camera was used to find the surface of the sample. A (x,y,z) translation stage was used to displace the sample. A combination of two aspheric lens (NA=0.3) was used to focus and collimate the incident and transmitted light.

2 Crystal symmetry from transmission measurement

Orientation dependent nonlinear absorption of helical light pulses in ZnO (11-20) and left-handed quartz (z-cut) crystal are shown in Fig. S2 for a specific displacements of the singularity. ZnO (11-20) has a cubic unit cell with 4-fold rotational symmetry and therefore shows a modulation with a periodicity of $\pi/2$. Quartz has a cubic unit cell with 6-fold rotational symmetry and therefore shows a modulation with a periodicity of $\pi/3$. Both crystals exhibit differential absorption between left- and right-handed asymmetrical helical light, defined as HD(Type I). The orientation dependence of transmission is independent of the position of the singularity. However, the magnitude of HD(Type I) signal remains invariant with crystal orientation. Similar results obtained for MgO (100) were presented in the main text.

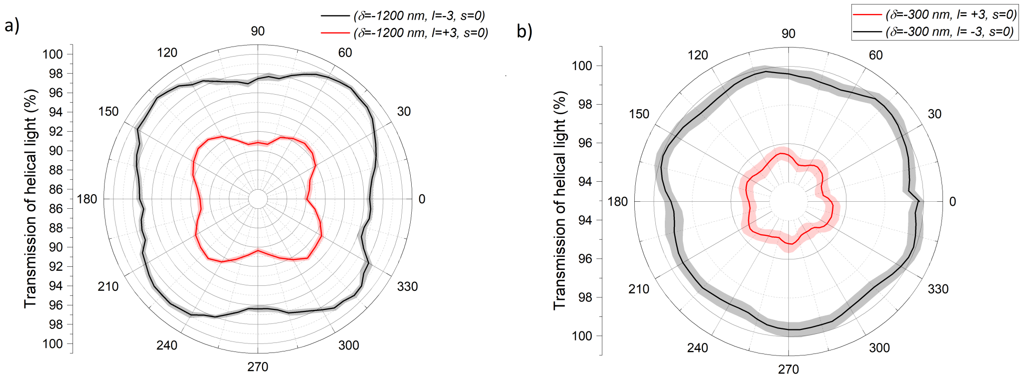

FIG. S 2. Orientational dependent transmission of helical light in crystal. For linearly polarized ($\varepsilon = 0.05$) asymmetrical OAM beam ($l = \pm 3$) in **(a)** ZnO (11-20) for a displaced singularity ($\delta = -1200$ nm) and **(b)** Left-handed α-quartz (z-cut) for a displaced singularity ($\delta = -300$ nm). The error bands represent the standard error at every position of the crystal for n=20.

The crystal structure can also be reproduced using linearly polarized Gaussian light pulses as shown in Fig. S3a for MgO (100) crystal, similar to the case of helical light (fig 1b-d). Therefore, the orientation dependent transmission is not limited to helical light. Fig. S3b shows the energy dependent transmission for a linearly polarized symmetric OAM beam ($l = +1, \delta = 0$) plotted as a function of crystal orientation. As the pulse energy is increased, the transmission of helical light decreased due to the increase in nonlinear absorption. For all three pulse energies above the onset for nonlinear absorption, the 4-fold symmetry is present.

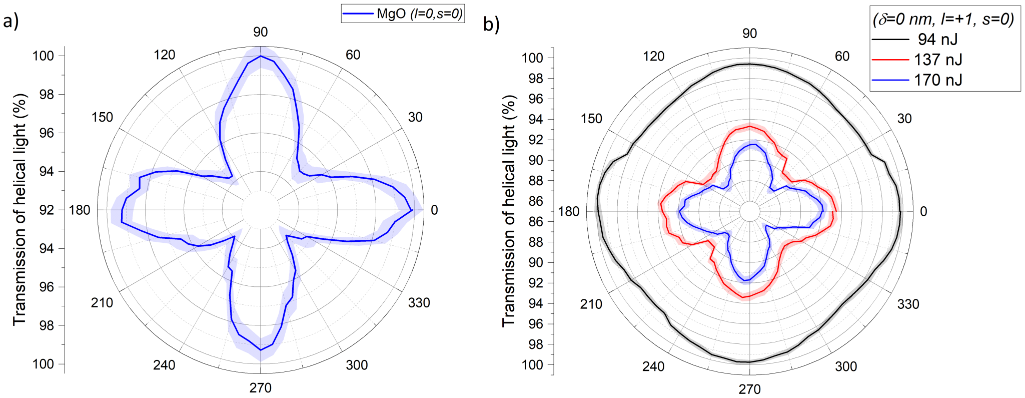

FIG. S 3. Angle dependent transmission of helical light in MgO (100). **(a)** For linearly polarized ($\varepsilon = 0.05$) Gaussian beam. **(b)** Energy dependent transmission as a function of crystal orientation for linearly polarized ($\varepsilon = 0.05$) symmetrical OAM beam ($l = \pm 3$). The error bands represent the standard error at every position of the crystal for n=20.

Fourier analysis of the orientation dependent transmission identifies the different modulation periodicities associated with the crystal symmetry. Figure S4 shows the Fourier power spectrum for the orientation dependent transmission curves of MgO (black) and quartz (blue). For MgO, single peak at 90° corresponds to a 4-fold symmetry. The quartz exhibits a dominant peak at 60 degrees and a smaller one at 120 degrees demonstrating the 6-fold symmetry. Stronger modulation depth in MgO than in quartz could be due to different crystal nonlinearities.

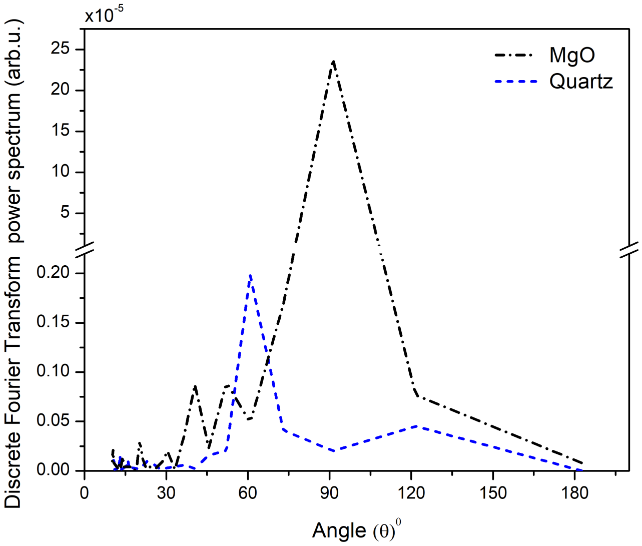

FIG. S 4. Discrete Fourier transform of the orientational dependent transmission in crystals. The black (blue) curve demonstrate the $\pi/2$ ($\pi/3$) periodicity in MgO (left-handed, z-cut, α-quartz).

3 Tunability and scalability of HD(Type I) in crystals

Figure S5 shows experimental results on control and tunability of HD(Type I) in MgO, obtained by (i) superimposing Gaussian and OAM beams, and (ii) varying the l-value. Similar to a-solids (Figure 5), the electric dipole-quadrupole coupling term is non-zero for asymmetric LG beams resulting in HD(Type I). Since this coupling term contains the gradient of the electric field it gives rise to l-dependence, both on its sign and value.

In c-solids, the MPAT process can still play a role, although ordered environment, both short and long range always ensures non-zero HD(Type I) signal upon isortropic averaging. In this case the intermediate states could be due to degenerate exciton states, defects, impurities, and boundary effects[3,4].

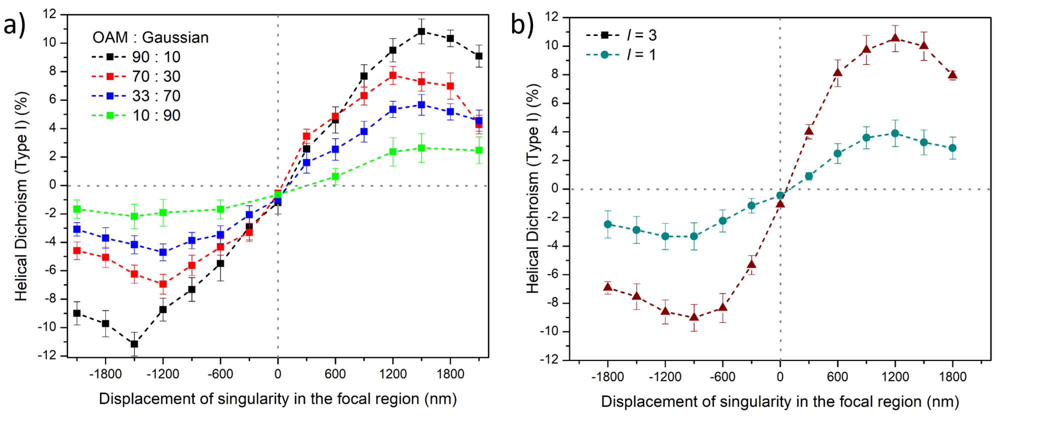

FIG. S 5. Tunability of Helical dichroism. Measured HD(Type I) in MgO as a function of the displacement of singularity for **a)** variable ratios of superposition of linearly polarized ($\varepsilon = 0.05$) OAM ($l = \pm 3$) and Gaussian beams, and **b)** linearly polarized ($\varepsilon = 0.05$) helical light with $l = \pm 1, \pm 3$. The error bars in **a)** and **b)** represent the standard error, of multiple measurements (n=3), calculated for an average peak fluence range used to obtain HD.

4 Fluence Calculation

In Laguerre-Gaussian beams with increasing l-value, the size of the null intensity region at the center increases. Consequently, for the same spot size higher pulse energies are required to reach the threshold for the onset of nonlinear absorption[5]. However, the peak fluence and intensity remains the same for all l-values as can be seen from the inset of Fig. 1a for l=3 and Fig.S6 for l=0.

From the threshold energies and peak power, the l-dependent peak fluence and the intensity was calculated based on the following equations[6]:

$$F_l\left[\frac{J}{cm^2}\right] = \frac{2^{(|l|+1)} r^{2|l|} e^{\frac{-2r^2}{w(z)^2}}}{|l|!\pi w(z)^{2(|l|+1)}} E_l\,[J] \qquad I\left[\frac{W}{cm^2}\right] = \frac{2^{(|l|+1)} r^{2|l|} e^{\frac{-2r^2}{w(z)^2}}}{|l|!\pi w(z)^{2(|l|+1)}} P_l\,[W] \tag{1}$$

where $l = 0; \pm 1; \pm 2; \pm 3 \ldots$ is the orbital angular momentum value, r is the radial direction, $w(z)$ is the radius of a beam evaluated at z and E_l is the threshold pulse energy, and P_l is the peak power for different l-values.

The peak fluence was obtained by evaluating the radial parameter r at the maxima of the intensity profile, which occurs when $r_l^2 = w_0^2|l|/2$. So, for a Gaussian beam ($l = 0$) $r = 0$, and at for OAM beam with $l = 1$ $r_1 = \pm\frac{w_0}{\sqrt{2}}$and so on. Substituting the above values for radial parameter, we obtain

$$\text{For } l = 0 \text{ beam} : F_0(r_0) = 2\frac{E_0}{\pi w_0^2} \tag{2}$$

$$\text{For l = 1 beam} : F_1(r_1) = 2e^{-1}\frac{E_1}{\pi w_0^2} \tag{3}$$

The l-dependent peak intensity was used to obtain the Keldysh parameter (eq - main text) and the spatial displacement x_0. Similarly, peak laser fluences or intensities were obtained for higher order OAM beams. In our experiments with different

l-values (Fig-1,4), HD was obtained by taking an average of differential absorption between left- and right- handed helical light over a peak fluence range after the the onset of nonlinear absorption. This insured that response regime we investigated remained the same.

5 Differential absorption with circulary polarized Gaussian beam

Circular dichroism (CD), defined as differential absorption of circularly polarized (CP) Gaussian beam, is not expected to be observed in amorphours solids. On the other hand, chiral solids such as quartz exhibit CD. However, solid-state CD signal is often overshadowed by signals arising from macroscopic anisotropies and linear birefringence[7], as can be seen in the figure below.

To discern the role of polarization and phase of light, we performed transmission measurements with CP Gaussian beams (l=0) in crystalline and amorphous solids. Figure S6 shows normalized transmission of a left- and right-CP Gaussian beam propagating through left-handed quartz and fused silica. In the absence of phase, the two curves overlap in the linear regime exhibiting no differential absorption. In the nonlinear regime, any signal fluctuations are within noise for both types of solids. Similar results were obtained in the case of MgO and right- handed quartz (not shown).

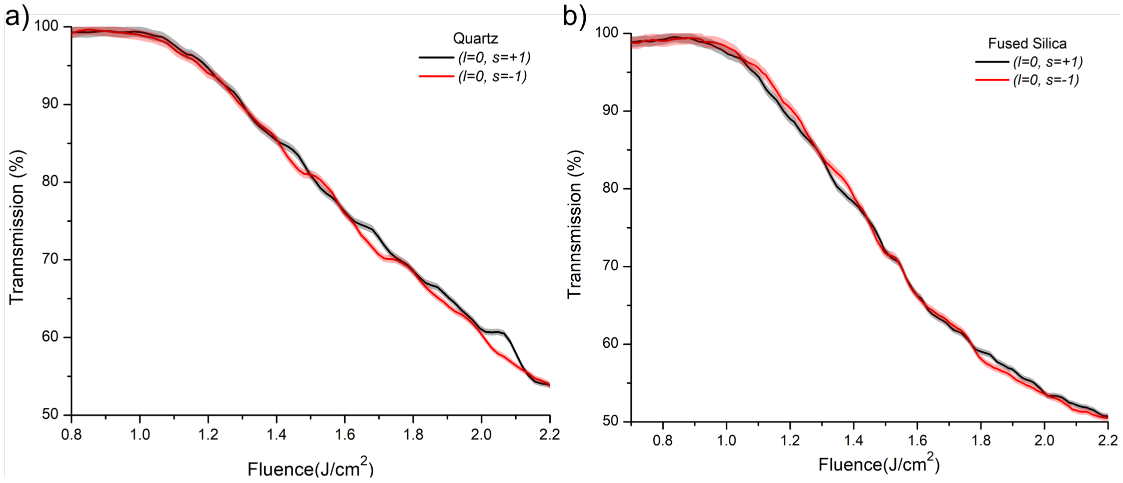

Fig S 6. Normalized transmission of Gaussian beams as a function of peak fluence in crystalline and amorphous solids. Transmission curves for left-circularly polarized ($s = +1$) and right-circularly polarized ($s = -1$) light in **a)** left-handed α-quartz and **b)** fused silica. The error bands represent the standard error for for n=3 independant measurements.

6 Noise check: Transmission measurements in air in the absence of sample

In order to check the background noise in our experimental setup, we performed transmission measurements conducted in air (absence of a sample). Such measurements enable us to ensure that the photodiodes detect identical signals when changing the helicity of the incoming linearly polarized light from $+l$ to $-l$. Fig. S7 a) shows transmission curves (ratio of PD_2/PD_1) of left (black) and right (red) handed asymmetrical LG beams through air. The red and black curves overlaps (within 0.5%) over the entire fluence range. In addition, Fig. S7 b) shows the difference between the PD1 (PD2) signal for linearly polarized $+l$ and $-l$. Signals are centered about zero with a fluctuation of $\pm 0.05V$ and curves almost overlap over the entire energy range. Both these results demonstrates that there is no arbitrary signal/noise introduced by the experimental setup to the observed differential absorption in crystalline and amorphous solids. Transmission measurements in air were performed before every experimental run and for every position of the displaced singularity.

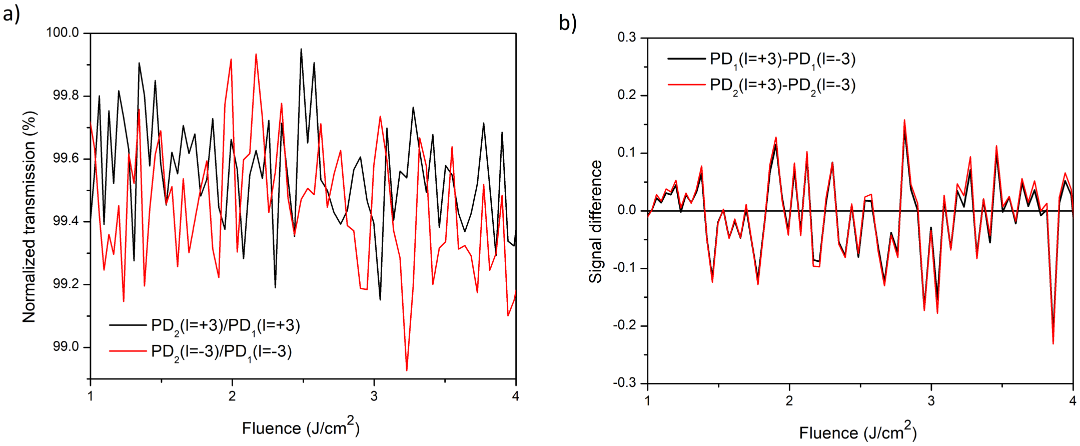

FIG. S 7. Transmission of linearly polarized ($\varepsilon = 0.05$) helical light in air for symmetrical OAM beam. a) Ratio of PD2 and PD1 signals for $l = +3$ (black) and $l = -3$ (red). **b**) The difference in photodiode signals for the two helicities, PD1 in black and PD2 in red. PD1 (PD2) monitors the incident (transmitted) light.

7 Asymmetric Laguerre-Gaussian beams

Asymmetric Laguerre-Gaussian beams denoted by an asymmetry parameter, δ, was used in our theoretical analysis. For arbitrary polarization in the paraxial regime, the field components can be written as[5]

$$u_o^{\pm}(x,y,z) = E_0 \exp[ikz] \left(\frac{\sqrt{2}\left((x \mp i\eta\delta) \pm i(y \mp i\zeta\delta)\right)}{w_0} \right)^{|l|} \exp\left(-\frac{(x^2+y^2)}{w_0^2}\right) L_{p-j}^{|(l+j)|}\left(\frac{2\rho^2}{w_0^2}\right) \tag{4}$$

$$\mathbf{E}^{\pm}(x,y,z) = \begin{cases} \alpha u_0^{\pm}(x,y,z) \\ \beta u_0^{\pm}(x,y,z) \\ if\left[(\alpha \pm i\beta)\frac{|l|w_0((x\mp i\eta\delta)\mp i(y\mp i\zeta\delta)}{(x\mp i\eta\delta)^2+(y\mp i\zeta\delta)^2} u_0^{\pm}(x,y,z) - \frac{2}{w_0}\left(\alpha(x)+\beta(y)\right) u_0^{\pm}(x,y,z)\right] \end{cases} \tag{5}$$

$$\mathbf{B}^{\pm}(x,y,z) = \begin{cases} -\beta \frac{k}{\omega} u_0^{\pm}(x,y,z) \\ \alpha \frac{k}{\omega} u_0^{\pm}(x,y,z) \\ if\frac{k}{\omega}\left[(\alpha \pm i\beta)\frac{|l|w_0((y\mp i\zeta\delta)\pm i(x\mp i\eta\delta))}{(x\mp i\eta\delta)^2+(y\mp i\zeta\delta)^2} u_0^{\pm}(x,y,z) - \frac{2}{w_0}\left(\alpha(y)-\beta(x)\right) u_0^{\pm}(x,y,z)\right] \end{cases} \tag{6}$$

where $f = \lambda/2\pi w_0$, ω is the laser frequency and $\rho = \sqrt{x^2+y^2}$. The $\pm$ represents the rotational direction of l with no radial node, $p = 0$. The polarization factors α and β are normalized such that $|\alpha|^2 + |\beta|^2 = 1$. Displacement of singularity in the x-y plane was achieved by varying η and ζ. For generalization, we substituted $\eta = 3/2$ and $\zeta = 1/8$, considering the movement of singularity to be perpendicular to the polarization following experimental conditions.

E_0 is the normalization factor obtained by integrating the intensity over all space (-∞ to + ∞), for ($l = 1$) it is given as :

$$E_0 = \sqrt{\frac{2k^2w_0^2}{\pi(\alpha^2+\beta^2)\left(2w_0^2+k^2w_0^4+2(\zeta^2+\eta^2)\delta^2\left(1+k^2w_0^2\right)\right)}} \tag{7}$$

Intensity profile of asymmetrical Laguerre-Gaussian beam with varying singularity position (δ) is shown in Fig. S8. As a correction to the paraxial regime (due to its finite l dependence) the longitudinal component of the field (E_z) can be considered[8,9]. The contribution of the longitudinal component becomes significant when light is focused tightly using a higher NA objective and is recently demonstrated in differentiating nanoparticle aggregates using helical light[10].

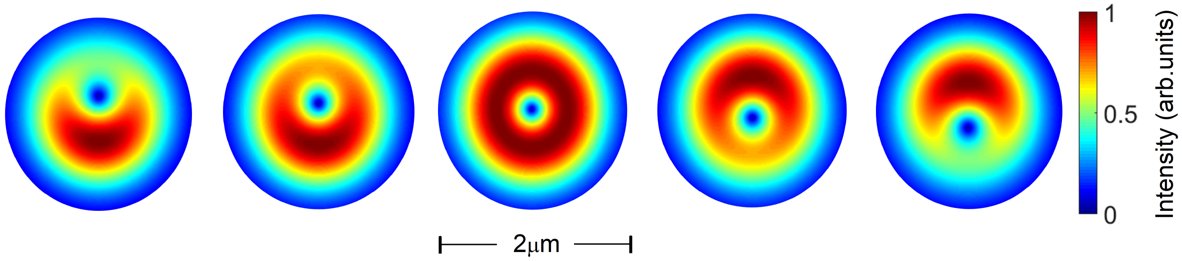

FIG. S 8. Asymmetrical Laguerre-Gaussian beam. Simulated intensity profiles of asymmetric OAM beams obtained by varying the δ parameter.

8 Multiphoton assisted tunneling

Absorption of electromagnetic field by any material results in electron transition from an unperturbed ground state to an excited state or continuum (conduction band in case of solids). The transition mechanism is primarily governed by the energy of the incident photon and energy difference between the ground and excited/continuum states. When photon energy is lower than the energy difference, absorption is dominated by nonlinear process either via multiphoton absorption when the incident field intensity is low or via tunneling when the field intensity is sufficiently large. The type of mechanism can be identified by Keldysh parameter, γ, for both solids and gases. When $\gamma \gtrsim 2$, transitions are dominated by multiphoton and when $\gamma < 1$ they are dominated by tunnelling[11–13]. $1 < \gamma < 2$ is the intermediate regime in which combination of both multiphoton and tunneling contribute to electron transitions, known as Multiphoton Assisted Tunneling (MPAT).

In MPAT, electron transitions from the ground state (valence band in solids) to an intermediate state (band tail/defect states in solids) by absorption of a single or multiple photons followed by transition to the continuum (conduction band in solids) via tunneling. MPAT is therefore responsible for the smooth and continuous transition from the dominant multiphoton ionization to the pure tunneling regime characterized by the varying Keldysh parameter. The idea of MPAT is based on the fact that whenever an incident photon is absorbed it causes the wavefunction to extend to larger distances depending on the wavelength and intensity of the incident light. Ionization via tunneling can readily occur from such extended and weakly bound states[11].

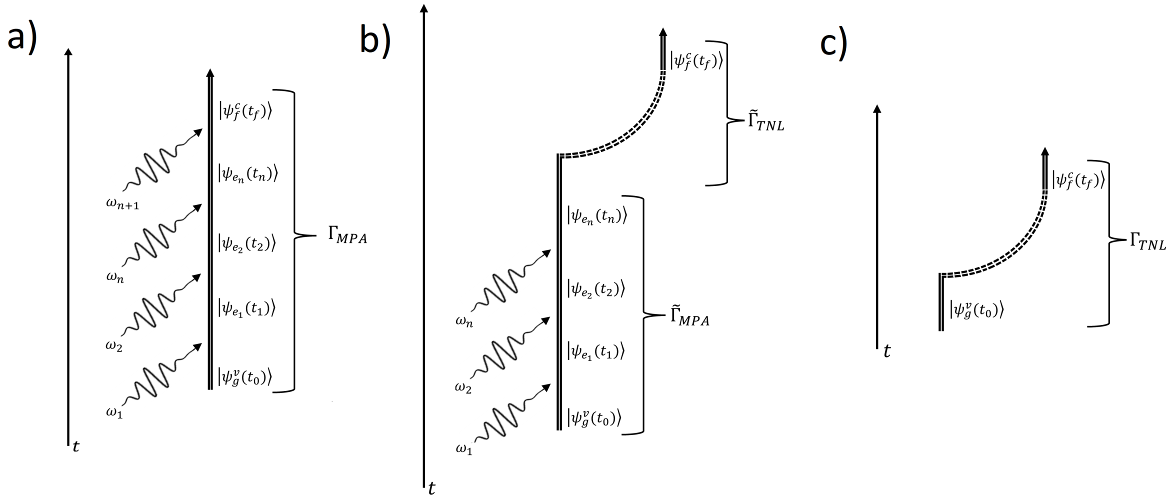

FIG. S 9. Feynman diagrams for a) multiphoton regime, it takes an absorption of $(n+1)\hbar\omega$ to reach the conduction band. b) MPAT process, absorption of n-photon leading to an excited/tail state and subsequent tunneling to the conduction band. c) tunneling regime, the incident field is strong enough to force the electron to tunnel from the valence band(ψ_g^v) to the conduction band(ψ_f^c).

Ionization probability for MPAT process can be qualitatively understood by a unified approach where the total probability amplitude consists of a product of two separate amplitudes corresponding to single/multiphoton absorption and tunneling. It can also be visually understood using Feynman diagram shown in Fig. S9 for all three different regimes. In general, transition

amplitude is defined as a projection of non-interacting state to a fully evolved state at large times[14–16] given by

$$M_{fi} = \lim_{t\to\infty}\langle\psi_f(t)|\Psi_i(t)\rangle \tag{8}$$

where $\Psi_i(t)$ is the total wave function of time-evolved ground state, ψ_g, when the laser field is turned on. $\Psi_i(t)$ is a solution of the time-dependent Schrodinger equation, $[i\hbar\frac{\partial}{\partial t} - \hat{H}(t)]\Psi_i(t) = 0$. In the presence of the laser field, the full Hamiltonian is given by $\hat{H}(t) = \hat{H}_0 + \hat{V}(t)$ where $\hat{H}_0$ is the field-free Hamiltonian and $\hat{V}(t)$ represents the interaction Hamiltonian of the system at time t. The final state $\psi_f(t)$, and initial state are orthogonal, $\langle\psi_f|\psi_g\rangle = 0$. The total wavefunction is obtained by solving the the time-dependent Schrodinger equation using the Green function $\hat{G}(t,t')$. The Green function is closely related to the time evolution operators, $\hat{G}(t,t') = -i\Theta(t-t')\hat{U}(t,t')$[14,16] where $\Theta(t-t')$ is the Heaviside step function and $\hat{U}(t,t')$ is a unitary time operator. Therefore, the total wavefunction can be expressed as

$$|\Psi_i(t)\rangle = \hat{U}_0(t,t_0)|\psi_g\rangle - \frac{i}{\hbar}\int_{t_0}^{t}\Theta(t-t')\hat{U}(t,t')V(t')|\psi_g(t')\rangle dt' \tag{9}$$

The first term corresponds to unperturbed evolution, $\hat{U}_0(t,t_0)$, of the ground state and the second term corresponds to evolution of the ground state when the laser is turned on at time t_0.

The transition amplitude is not limited to just initial and final states. We can partition the total Hamiltonian to introduce intermediate excited states, which can be obtained by expanding the time propagator $\hat{U}(t,t')$ in terms of the Dyson series[14–17]. In the Dyson expansion, we consider up to the second order term which corresponds to first order MPAT process (a single photon absorption to the intermediate state and subsequent tunnelling from this intermediate state to the continuum/conduction band). By substituting the expanded time propagator into the total wavefunction (eq. 9) and afterwards inserting this expanded wavefunction into the transition amplitude (eq. 8) we obtain

$$M_{fg} = \frac{1}{i\hbar}\int_{t_0}^{t}\langle\psi_f(\tau_1)|\hat{V}_i(\tau_1)|\psi_g(\tau_1)\rangle d\tau_1 + \frac{1}{\hbar^2}\int_{t_0}^{t}\int_{t_0}^{\tau}\langle\psi_f(\tau)|\hat{V}_f(\tau)\hat{U}(\tau,\tau_1)\hat{V}_i(\tau_1)|\psi_g(\tau_1)\rangle d\tau_1 d\tau \tag{10}$$

where $\hat{V}_i$ and $\hat{V}_f$ are the interaction Hamiltonian at time τ_1 and τ, respectively. Also, we used the property of the Heaviside step function $\lim_{t\to\infty}\Theta(t-\tau) = 1$. The first term in the above equation represents standard ground state tunneling and the second term represents the first order MPAT process. When experimental intensities are not sufficient for ground state tunneling (intermediate regime, $1 \lesssim \gamma \lesssim 2$) the second term becomes dominant. Therefore, for a multitude of intermediate states, the MPAT transition amplitude is given by

$$M_{fg} = \frac{1}{\hbar^2}\sum_m\int_{t_0}^{t}\int_{t_0}^{\tau}\langle\psi_f(\tau)|\hat{V}_f(\tau)|\psi_m(\tau)\rangle\langle\psi_m(\tau)|\hat{U}(\tau,\tau_1)\hat{V}_i(\tau_1)|\psi_g(\tau_1)\rangle d\tau_1 d\tau \tag{11}$$

where we introduced the projection of the intermediate state $\mathbb{1} = \sum_m|\psi_m(\tau)\rangle\langle\psi_m(\tau)|$[18]. Using the property $\langle\psi_m(\tau)|\hat{U}(\tau,\tau_1) = \langle\psi_m(\tau_1)|$, and explicitly writing the time component we obtain

$$M_{fg} = \frac{1}{\hbar^2}\sum_m\int_{t_0}^{t}\langle\psi_f(\tau)|\hat{V}_f(\tau)|\psi_m(\tau)\rangle\int_{t_0}^{\tau}\langle\psi_m|\hat{V}_i(\tau_1)|\psi_g\rangle e^{i\omega_{mg}\tau_1}d\tau_1 d\tau \tag{12}$$

The multiphoton component of the interaction Hamiltonian, $\hat{V}_i$, can be written in a non-dipole form containing E1, M1 and E2 terms. For single photon absorption, we substitute $\hat{V}_i(t') = \hat{V}_i e^{-i\omega t'}$ in the above equation (where ω is the laser frequency) to get

$$M_{fg} = \sum_m \underbrace{\frac{\langle\psi_m|\hat{V}_i|\psi_g\rangle}{\hbar(\omega_{mg}-\omega)}}_{M_{SPA}}\underbrace{\frac{i}{\hbar}\int_{t_0}^{t}d\tau\langle\psi_V(\tau)|\hat{V}_f(\tau)|\psi_m(\tau)\rangle e^{i(\omega_{mv}-\omega)\tau}}_{M_{TNL}} \tag{13}$$

The transition amplitude M_{fg} is a product of time-independent transition amplitude, M_{SPA}, corresponding to a single photon absorption to an intermediate state and time-dependent amplitude, M_{TNL}, corresponding to tunneling from intermediate state to continuum/conduction band. $\hat{V}_i$ ($\hat{V}_f$) is the multiphoton (tunneling) interaction Hamiltonian.

For the nth order MPAT process, the transition amplitude consists of a product of the n-photon absorption and subsequent tunneling.

$$M_{fg} = \left(\frac{1}{i\hbar}\right)^2 \sum_m \sum_l \int_{t_0}^{t} d\tau \langle \psi_f(\tau)|\hat{V}_f(\tau)|\psi_m(\tau)\rangle \int_{t_0}^{\tau} dt' \langle \psi_m|\hat{V}\left(t'\right)|\psi_l\rangle c_l^{(n-1)}\left(t'\right) e^{i\omega_{ml}t'} \tag{14}$$

where $c_l^{(n-1)}(\tau)$ is the probability amplitude of the $(n-1)^{\text{th}}$ order perturbation (multiphoton probability amplitude)[19]. The nth order probability amplitude can be expressed as

$$c_m^{(n)}(\tau) = \left(\frac{1}{i\hbar}\right) \sum_l \int_{t_0}^{\tau} dt' \langle \psi_m|\hat{V}\left(t'\right)|\psi_l\rangle c_l^{(n-1)}\left(t'\right) e^{i\omega_{ml}t'} \tag{15}$$

Multiphoton transitions in MPAT will result in multitiude of excited state cross-correlation terms and coupling terms arising from multipole expansion due to the tensor nature of the material response. However, the material cross terms (such as electric–magnetic dipole and electric dipole–quadrupole coupling) will have similar qualitative behaviour as that of a single photon case. Therefore, to keep the equations tractable, we consider the MPAT process using single photon absorption in the manuscript.

Equations 12 can also be obtained by writting the time evolved initial state $\psi_g(\tau)$ as a linear combination of eigenstates (bound-state) of the unperturbed Hamiltonian[19,20], as

$$|\psi_g^{(n)}(\mathbf{r},\tau)\rangle = \sum_m c_m^{(n)}(\tau) e^{-i\frac{E_m}{\hbar}\tau}|\psi_m(\mathbf{r})\rangle = \sum_m c_m^{(n)}(\tau)|\psi_m(\mathbf{r},\tau)\rangle \tag{16}$$

where E_m is the energy of the mth eigenstate, $\psi_m(\mathbf{r})$, where $\omega_{ml} = (E_m - E_l)/\hbar$

9 Total MPAT transition rate for linear polarization

To obtain a numerical estimate of the total transition rate, for simplicity, we consider the incident field as linearly polarized. Therefore the probability amplitude from the main text (equation 11) becomes:

$$M_{cv} = \frac{iA_0 e}{m\hbar(2\pi)^{3/2}} \frac{1}{\gamma_0} \sum_m \mathrm{p}_{mv}^{SPA} \frac{p_{cm}}{k_x} \sum_{\eta=-\infty}^{\infty} \eta \mathrm{J}_\eta(-\gamma_0 k_x) \left[\lim_{T\to\infty} \frac{2\hbar sin\left((E_g+\varepsilon-n\hbar\omega)\frac{T}{2\hbar}\right)}{(E_g+\varepsilon-n\hbar\omega)}\right] \tag{17}$$

where $\varepsilon = \frac{\hbar^2 k^2}{2m^*}$ is the total kinetic energy, $m^{*-1} = m_1^{-1} - m_0^{-1}$ is the total effective electron mass and $n\hbar\omega = \eta\hbar\omega + \hbar\omega$, and assuming the intermediate and conduction band states have similar $\mathbf{k}$ ($\mathbf{k}'' \approx \mathbf{k}' \approx \mathbf{k}$) the material response $p_{cm} = \int_{-\infty}^{\infty} u_{\mathbf{k}}^{c*}(\mathbf{r}) p_x u_{\mathbf{k}''}^{m}(\mathbf{r}) d^3r$

Following the main text, we further define the tunneling transition contribution as

$$\mathrm{p}_{cm}^{TNL} = \frac{m_1}{m\hbar(2\pi)^{3/2}} \frac{p_{cm}}{k_x} \sum_{\eta=-\infty}^{\infty} \eta \mathrm{J}_\eta(-\gamma_0 k_x) \tag{18}$$

Using the property $\lim_{T\to\infty} T^2 \frac{sin^2(x)}{x^2} = 2\pi\hbar T \delta\left(\varepsilon + E_g - n\hbar\omega\right)$ where $x = (E_g+\varepsilon-n\hbar\omega)\frac{T}{2\hbar}$[19], the transition rate from valence to the conduction band, defined as $W_{cv} = \frac{d}{dt}|M_{cv}|^2$, can be expressed as

$$W_{cv}(\mathbf{k}) = 2\pi\hbar\omega^2 \sum_m \left|\mathrm{p}_{cm}^{TNL}\right|^2 \left|\mathrm{p}_{mv}^{SPA}\right|^2 \delta\left(\varepsilon + E_g - n\hbar\omega\right) \tag{19}$$

The total transition rate (per unit of volume) can be obtained by integrating over crystal momentum associated with the discrete transition:

$$W = \int \frac{\mathrm{d}^3 k}{(2\pi)^3} W_{cv}(\mathbf{k})$$

Using the properties of Bessel's functions, the weak-field limit for the non-perturbative discrete transition rate is given by[21,22]

$$W = \frac{1}{(2\pi)^4\hbar}\sum_m \left(\frac{m_1\omega p_{cm}}{m(\eta-1)!}\right)^2 \left|p_{mv}^{SPA}\right|^2 \left(\frac{eA_0}{2m_1\omega}\right)^{2\eta} \left(\frac{2m^*}{\hbar^2}\right)^{(2\eta+1)/2} \left(\frac{(n\hbar\omega - E_g)}{(2\eta-1)}\right)^{(2\eta-1)/2} \tag{20}$$

where

$$\left|p_{mv}^{SPA}\right|^2 = \frac{1}{\hbar^2(\omega_{mv}-\omega)^2}\left[|\mu_i^{mv}|^2|E_i^{\pm}|^2 + |m_i^{mv}|^2|B_i^{\pm}|^2 + 2\langle\mu_i^{mv} m_i^{vm}\rangle_P \mathrm{Im}\left[E_i^{\pm*}B_i^{\pm}\right] + \frac{2}{3}\langle\mu_i^{mv}\theta_{ij}^{vm}\rangle_P \mathrm{Re}\left[\nabla_i E_j^{\pm} E_i^{\pm*}\right]\right] \tag{21}$$

Difference in the interband transition rates between left- and right-handed helical light with beam asymmetry parameter δ can be expressed as:

$$\Delta\mathbf{W} = W_{\delta}^{+} - W_{\delta}^{-} = \frac{1}{\hbar^2(\omega_{mv}-\omega)^2}\tilde{W}\left[\Omega\left(\frac{2}{3}\mu_i^{mv}\theta_{ij}^{vm}\right)\left(\mathrm{Re}\left[\nabla_i E_j^{+} E_i^{+*}\right] - \mathrm{Re}\left[\nabla_i E_j^{-} E_i^{-*}\right]\right)\right] = D\left(\Upsilon^{+} - \Upsilon^{-}\right) \tag{22}$$

HD(Type I) can now be defined in terms of energy absorbed Γ normalized with respect to incident energy, $\mathscr{E}_{inc}$ as:

$$\Delta\Gamma = \underbrace{\Gamma^{+} - \Gamma^{-}}_{\text{HD (Type I)}} = \frac{\hbar}{\mathscr{E}_{inc}}\left(W_{\delta}^{+} - W_{\delta}^{-}\right) = \mathscr{D}\left(\Upsilon^{+} - \Upsilon^{-}\right) \tag{23}$$

where $W = \left|p_{mv}^{SPA}\right|^2\tilde{W}$ from eq-29, $\mathscr{D} = \frac{\hbar D}{\mathscr{E}_{inc}}$ and $\Upsilon^{\pm} = \mathrm{Re}\left[\nabla_i E_j^{\pm} E_i^{\pm*}\right]$ represent the optical helicity term describing the handedness of helical light. Optical helicity can be expanded as[23], $\Upsilon^{\pm} = \left[\left(\nabla_\alpha E_\beta E_\alpha^* + E_\alpha(\nabla_\alpha E_\beta)^\dagger\right)\right] = \mathrm{Re}\left[E_i^{\pm*}\nabla_i E_j^{\pm\dagger}\right]$. The above equation was plotted in Fig.5 as a function of displacement of singularity in the main text.

10 Supplementary References

1. Marrucci, L., Manzo, C., & Paparo, D., Optical Spin-to-Orbital Angular Momentum Conversion in Inhomogeneous Anisotropic Media. *Phys. Rev. Lett.*, **96**, 163905 (2006).
2. Hugo, L., et al. Arbitrary optical wavefront shaping via spin-to-orbit coupling. *J. Opt*. **18**, 124002 (2016).
3. Lee, C.C., & Fan, H.Y. Two-photon absorption with exciton effect for degenerate valence bands *Phys. Rev. B* **9**, 3502 (1974).
4. Gnani, E., et al. Band-structure calculations of SiO_2 by means of Hartree-Fock and Density-Functional Techniques *IEEE transactions on electron devices* **47**, no. 10 (2000)
5. Bégin, JL., Jain, A., & Parks, A. et al. Nonlinear helical dichroism in chiral and achiral molecules. *Nat. Photon.* **17**, 82–88 (2023).
6. Hnatovsky, C., et al. Materials processing with a tightly focused femtosecond laser vortex pulse. *Opt. Lett.* 35 **20**, 3417 (2010).
7. Kuroda, R., Novoa, J.J., Chirality in crystals. Engineering of crystalline materials properties, *Springer*; New York. p. 251 (2008)
8. Cerjan, A., et al. Orbital angular momentum of Laguerre-Gaussian beams beyond the paraxial approximation. *J. Opt. Soc. Am.A* **28**, 2253-2260 (2011).
9. Forbes, K.A., et al. Relevance of longitudinal of paraxial optical vortices *J. Opt.* **23**, 075401 (2021).
10. Brullot, W., et al. Resolving enantiomers using the optical angular momentum of twisted light. *Sci. Adv.* **2**, 150134 (2021).
11. Klaiber, M. & J.S. Briggs Crossover from tunneling to multiphoton ionization of atoms. *Phys. Rev. A.* **94**, 053405 (2016)
12. Schaffer, C.B., et al. Laser-induced breakdown and damage in bulk transparent materials induced by tightly focused femtosecond laser pulses *Meas. Sci. Technol.* **12**, 1784 (2002)
13. Wu, A.Q., et al. Femtosecond laser absorption in fused silica: Numerical and experimental investigation *Phys. Rev. B* **72**, 085128 (2005)
14. Long, Z.J. & Liu, W-K. Keldysh theory of strong-field ionization *Canadian journal of Physics* **88**, 4, (2010)

15. Becker, A. & Faisal F.H.M. Intense-field many-body S-matrix theory *J. Phys. B: At. Mol. Opt. Phys.* **38** (2005)

16. Reiss, H.R. Theoretical methods in quantum optics: S-matrix and Keldysh techniques for strong-field processes *Prog. Quant. Electr.* **16**, 1-71 (1992)

17. Becker, W. et al. Above-Threshold Ionization: From Classical Features to Quantum Effects. *Advances In Atomic, Molecular, and Optical Physics, Academic Press,* **48** (2002).

18. Sakurai, J., & Napolitano, J. *Modern Quantum Mechanics (2nd ed.)* Cambridge University Press (2017).

19. Boyd, R.W., *Nonlinear Optics (3rd ed.)* (Academic Press, 2020).

20. Serebryannikov, E.E., & Zheltikov, A.M., Strong-Field Photoionization as Excited-State Tunneling *Phys. Rev. Lett.* **116**, 123901, (2016)

21. Brandi, H.S. & de Araujos, C.B., Multiphoton absorption coefficients in solids: a universal curve. *J. Phys. C: Solid State Phys.* **16** 5929 (1983)

22. Brandi, H.S., Davidovich, L., & Zagury N., High-intensity approximations applied to multiphoton ionization. *Phys. Rev. A* **24**, 2044 (1981)

23. Bégin, J.L., Jain, A., & Parks, A. et al. Nonlinear helical dichroism in chiral and achiral molecules. *Nat. Photon.* **17**, 82–88 (2023).
